# Spent Coffee Grounds Derived Carbon Loading C, N Doped TiO_2_ for Photocatalytic Degradation of Organic Dyes

**DOI:** 10.3390/ma16145137

**Published:** 2023-07-21

**Authors:** Yanling Jin, Jiayi Wang, Xin Gao, Fang Ren, Zhengyan Chen, Zhenfeng Sun, Penggang Ren

**Affiliations:** 1 Faculty of Printing, Packaging Engineering and Digital Media Technology, Xi’an University of Technology, Jinhua South Road No. 5, Xi’an 710048, China; jinyl@xaut.edu.cn (Y.J.); gx19834269248@163.com (X.G.);; 2 School of Materials Science and Engineering, Xi’an University of Technology, Jinhua South Road No. 5, Xi’an 710048, China

**Keywords:** titanium dioxide, spent coffee grounds–derived carbon, C, N doping, photocatalytic degradation, organic dyes, dye mixture

## Abstract

Titanium dioxide (TiO_2_) is an ideal photocatalyst candidate due to its high activity, low toxicity and cost, and high chemical stability. However, its practical application in photocatalysis is seriously hindered by the wide band gap energy of TiO_2_ and the prone recombination of electron-hole pairs. In this study, C, N doped TiO_2_ were supported on spent coffee grounds-derived carbon (ACG) via in situ formation, which was denoted as C, N–TiO_2_@ACG. The obtained C, N–TiO_2_@ACG exhibits increased light absorption efficiency with the band gap energy decreasing from 3.31 eV of TiO_2_ to 2.34 eV, a higher specific surface area of 145.8 m^2^/g, and reduced recombination rates attributed to the synergistic effect of a spent coffee grounds-derived carbon substrate and C, N doping. Consequently, the optimal 1:1 C, N–TiO_2_@ACG delivers considerable photocatalytic activity with degradation efficiencies for methylene blue (MB) reaching 96.9% within 45 min, as well as a high reaction rate of 0.06348 min^−1^, approximately 4.66 times that of TiO_2_ (0.01361 min^−1^). Furthermore, it also demonstrated greatly enhanced photocatalytic efficiency towards methyl orange (MO) in the presence of MB compared with a single MO solution. This work provides a feasible and universal strategy of synchronous introducing nonmetal doping and biomass-derived carbon substrates to promote the photocatalytic performance of TiO_2_ for the degradation of organic dyes.

## 1. Introduction

During recent decades, the environmental pollution issue, especially the water pollution issue, has emerged as an important concern with a significant impact on humans’ daily lives [1]. In particular, organic dyes, which possess a complex molecular structure and good chemical stability, are challenging to biodegrade naturally [2,3]. Plentiful technologies including adsorption [4,5], biodegradation [6,7], and membrane [8], etc. have been utilized to separate or remove organic pollutants from wastewater, but cannot degrade them thoroughly and eventually make them enriched in living organisms [9,10]. Therefore, it is critically important to develop a highly efficient and eco-friendly strategy to solve these environmental issues.

In this context, photocatalytic degradation is regarded as an emerging and green technology that converts renewable, clean, and freely available solar energy to chemical energy to degrade pollutions into more benign species without secondary pollution [11,12,13,14,15,16]. As one of the most promising photocatalysts, titanium dioxide (TiO_2_) has drawn substantial attention due to its high activity, low toxicity and cost, and high chemical stability [11,16,17,18,19,20]. However, there are two main intrinsic shortcomings of TiO_2_: one is its relatively wide band gap (about 3.2 eV), meaning that only UV light (less than 5% of the total solar spectrum) can be absorbed and efficiently utilized [21]; the other is the high recombination rate of the photogenerated electron-hole (e^−^-h^+^) pairs, leading to low quantum efficiency [22,23]. Therefore, it is imperative that we develop TiO_2_-based photocatalysts with high light utilization and the extended lifetime of photogenerated carriers.

Currently, great efforts have been made to regulate the band gap of TiO_2_ through producing donor or acceptor states in the band gap of TiO_2_ [24,25,26]. The most investigated strategy is doping with metallic or nonmetallic elements [27]. However, considering that metal-doped TiO_2_ usually suffers from poor thermal stability or photocorrosion [28,29], coupled with the toxicity and cost of the metal doping, non-metal element doping, such as N, C, S, F or B, is more favorable and shows great potential in enhancing visible-light responsive activity [30,31]. In the TiO_2_ lattice, N doping will easily bring in the O-Ti-N structure through the combination of the O 2p orbital with N 2p orbitals, thus introducing an impurity energy level with a lower maximum valence band (VB) potential than that of TiO_2_ [32,33,34,35,36,37]. It has been reported that C doping can efficiently narrow the band gap through forming various impurity energy levels between the band gaps [38,39,40]. Furthermore, compared to single non-metal doping, double-doping has demonstrated a more appreciable effect [33,41].

Combining TiO_2_ with carbon material is proven to be a versatile strategy for inhibiting the rapid compounding of electron-hole pairs [42,43,44,45,46]. Carbon material with excellent electrical conductivity can transfer the photogenerated electrons on the conduction band quickly and prevent the recombination of photogenerated electron holes [47,48]. In addition, the porous nature of carbon materials can provide a good carrier for TiO_2_ and increase the adsorption performance, which can enrich the pollutants inside and outside the pores and facilitate subsequent rapid photocatalytic degradation [49,50]. Biomass carbon materials are widely sourced in nature and have natural morphology and a pore structure, which can be applied by simple treatment [51,52]. Therefore, biomass can be used as an excellent carbon precursor for fabricating TiO_2_/carbon composite photocatalysts [53]. Coffee has become an indispensable beverage in many parts of the world, and large amounts of coffee grounds are concomitantly generated. The rational utilization of spent coffee grounds through converting them to a new resource will be significant [54,55,56]. In our previous work, spent coffee grounds-derived carbon was applied for electromagnetic interference shielding [57] and the adsorption of organic dyes [4], supercapacitors [58], and photocatalysis [20].

In this study, spent coffee grounds-derived carbon supported C, N doped TiO_2_ (C, N–TiO_2_@ACG) photocatalysts were prepared using tetrabutyl titanate as the titanium source and a carbon source, nitric acid, as the nitrogen source. The obtained C, N–TiO_2_@ACG exhibits increased light absorption efficiency, a higher specific surface area, and reduced recombination rates. Consequently, it delivers excellent photocatalytic degradation performance for single MB and mixed dyes of MO and MB (MOMB). This work provides a reference and extension method for the further development of TiO_2_–based photocatalysts and high value-added utilization of coffee grounds.

## 2. Experiments

### 2.1. Materials

Tetrabutyl titanate (TBOT), ethanol, nitric acid, and hydrochloric acid (HCl) were purchased from the Sinopharm Chemical Reagent Co., Ltd. (Beijing, China). Coffee grounds were purchased in a local coffee shop (Xi’an, China). Methylene blue (MB) and Methyl orange (MO) were purchased from the Aladdin Reagent Co., Ltd. TiO_2_ nano-particles were purchased from Sigma Aldrich (St. Louis, MO, USA). All chemicals and reagents except the coffee grounds were of analytical grade and used without further purification.

### 2.2. Preparation of Coffee Grounds-Derived Carbon

The coffee grounds were smashed and sieved discretely. They were then soaked in 0.2 M dilute hydrochloric acid for 4 h, washed with ethanol and deionized water to neutral, and dried under a vacuum at 60 °C for 24 h. Subsequently, the coffee grounds were pre-carbonized at 1000 °C for 2 h in an N_2_ atmosphere, soaked in 60 g/L KOH solution for 4 h for activation with the ratio of KOH:coffee grounds = 1:1, followed by being dried at 80 °C for 8 h and carbonized again under the same conditions. Finally, the obtained coffee grounds-derived carbon was washed with deionized water and dried, which is denoted as ACG.

### 2.3. Preparation of C, N–TiO_2_@ACG

**Figure 1:**
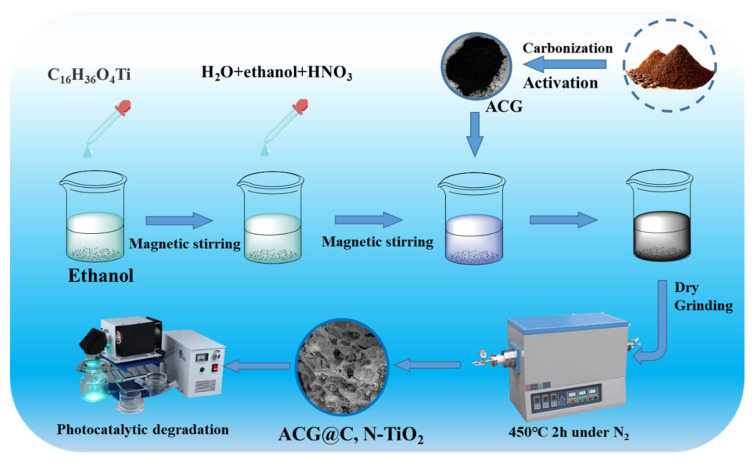
Schematic illustration of the preparation of C, N-TiO_2_@ACG and its application to photocatalytic degradation.

As illustrated in Figure 1, first, 10 mL of tetrabutyl titanate solution was added dropwise to 50 mL of absolute ethanol and stirred for 30 min to obtain a homogeneous solution (solution A). Next, 3 mL of deionized water, 3 mL of anhydrous ethanol, and 1 mL of 65% nitric acid solution were mixed thoroughly to produce solution B, which was slowly dropped into solution A. At this time, 1 g of ACG was slowly added to the mixed solution and magnetically stirred for 6 h to obtain the gel. After drying and grinding, the powder was calcined at 450 °C for 2 h in an N_2_ atmosphere. The obtained photocatalysts with the mass ratios of TBOT to ACG at 1:0.5, 1:1 and 1:2 were labeled as 1:0.5 C, N–TiO_2_@ACG, 1:1 C, N–TiO_2_@ACG and 1:2 C, N-TiO_2_@ACG, respectively. For comparison, C, N–TiO_2_ was also prepared under the same conditions without adding ACG. The commercial nano–TiO_2_ was labeled TiO_2_.

### 2.4. Materials Characterizations

The crystal structure was characterized by X-ray diffraction (Shimadzu XRD-7000, Kyoto, Japan) with Cu Kα radiation (λ = 0.15418 nm). The morphologies were observed on a field emission SEM (Hitachi S-450, Japan) with an accelerating voltage of 15 kV. The Raman spectra of the photocatalysts were analyzed using a Raman spectrometer (Shanghai Precision Instrument Co., Ltd. GS1000, Shanghai, China). The Brunauer–Emmett–Teller (BET) technique was used to assess the specific surface areas and porosities of the photocatalysts on a surface area analyzer (MicrotracBel mike2020, Osaka, Japan). The electrochemical impedance spectroscopy (EIS) was tested on an electrochemical analyzer (Chenhua Instruments Company CHI 760B, Shanghai, China) in the frequency range of 0.01–100,000 Hz. The ultraviolet-visible diffuse reflectance spectra (DRS) were obtained with a UV-vis diffuse reflectance spectrophotometer (HITACHI U3310, Oita, Japan). Photoluminescence (PL) spectra of the photocatalysts were conducted using a fluorescence spectrophotometer (RF-6000) at the excitation wavelength of 380 nm.

### 2.5. Photocatalytic Degradation

The photocatalytic degradation reaction was implemented with MB and MO solution as the organic pollutants irradiated vertically by a 300-W xenon lamp (PLS-SXE300/300UV xenon lamp (Perfect Light Co., Beijing, China)). Typically, 100 mg of the photocatalyst was dispersed in 100 mL of 20 mg/L organic pollutant solution (MB or MO solution). Before irradiation, the suspension was stirred for 30 min in the dark to achieve adsorption equilibrium. When degrading the mixed solution of MB and MO, 50 mL of 20 mg/L aqueous solution of MB and MO was mixed and 50 mg of photocatalyst was added for photocatalytic degradation. At regular time intervals (15 min), 1.0 mL suspension was withdrawn and analyzed by UV-vis spectrophotometer. The degradation rate (Dr) of the organic pollutant (MB or MO) is calculated in Equation (1) as follows:

(1) $$Dr=1-\frac{C}{C_{0}}$$

where C_0_ and C refer to the initial contaminant concentration and the contaminant concentration at the test time, respectively.

## 3. Results and Discussion

**Figure 2:**
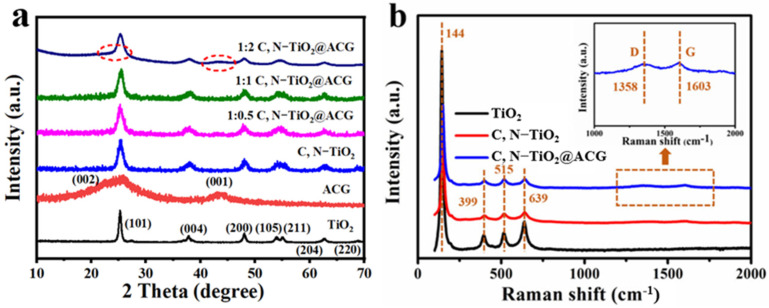
XRD patterns of photocatalysts (**a**), Raman spectra (**b**) of TiO_2_, C, N–TiO_2_ and 1:1 C, N–TiO_2_@ACG.

The XRD patterns shown in Figure 2a display that for TiO_2_, C, N–TiO_2_ and C, N–TiO_2_@ACG, there are diffraction peaks at 25.2°, 37.0°, 37.8°, 48.1°, 53.9°, 55.1° and 62.8°, which can be indexed to the (101), (103), (004), (200), (105), (211) and (204) crystalline planes of typical anatase–TiO_2_. These results indicate that the structure is not changed by the addition of ACG. For ACG, the broad diffraction peaks appearing at 25.6° and 43.4°correspond to the (002) and (001) crystal planes of graphitized carbon. For the C, N–TiO_2_@ACG, the XRD patterns are largely consistent with that of TiO_2_. When the ratio of ACG is low, no obvious diffraction peaks of carbon can be observed due to its low crystallinity and its surface covering by TiO_2_ particles [20]. With the increase in ACG, the diffraction peaks belonging to carbon gradually emerge and become obvious, as marked in 1:2 C, N–TiO_2_@ACG with a red circle, demonstrating the successful recombination of TiO_2_ and ACG. 

The Raman spectra of TiO_2_, C, N–TiO_2_ and 1:1 C, N–TiO_2_@ACG composite catalysts are shown in Figure 2b. The characteristic peaks at 144, 399, 515 and 639 cm^−1^ in all samples belong to TiO_2_, which is consistent with the XRD results. Furthermore, two reflections located at 1358 cm^−1^ and 1603 cm^−1^ in 1:1 C, N–TiO_2_@ACG are assigned to the D (disordered carbon) and G (graphitized carbon) bands of the carbon from the ACG. The calculated I_D_/I_G_ intensity ratio was 0.9, confirming the high graphitization degree of the ACG, which means that such highly graphitized carbon possesses excellent electronic conductivity and can enhance the separation efficiency of carriers in photocatalytic reactions. Furthermore, the weaker intensity of the characteristic peaks of carbon is probably associated with the loading of TiO_2_ on the ACG.

**Figure 3:**
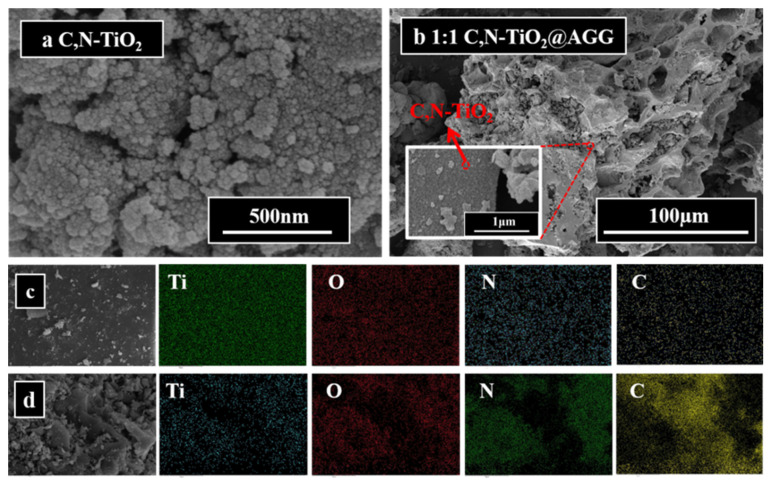
SEM images (**a**) of C, N–TiO_2_, (**b**) C, N–TiO_2_@ACG, element mapping of (**c**) C, N–TiO_2_, (**d**) 1:1 C, N–TiO_2_@ACG.

The morphologies of photocatalysts were characterized by SEM and are shown in Figure 3. The SEM image of C, N–TiO_2_ in Figure 3a shows that C, N–TiO_2_ appears as nano-sized particles with a slight agglomeration, which is caused by the van der Waals force between particles [59]. As shown in Figure 3b and Figure S1a,b, C, N-TiO_2_@ACG exhibits a well-constructed macroscale porous architecture, which is naturally derived from coffee grounds and the different amounts of C, N–TiO_2_ particles that are uniformly loaded on the surface of the carbon skeleton of the ACG with the change in ratio, as for 1:0.5 C, N–TiO_2_@ACG, 1:1 C, N–TiO_2_@ACG and 1:2 C, N–TiO_2_@ACG; this can be further demonstrated by the high-magnification image of 1:1 C, N–TiO_2_@ACG illustrated in the inset of Figure 3b. This unique natural porous structure is conducive to the increase in the specific surface area of the catalyst, enhancing its adsorption of organic pollutants, and thus improving the photocatalytic performance. Element mapping (Figure 3c) shows that Ti, O, C and N were evenly distributed in C, N–TiO_2_, indicating that C and N elements were successfully doped into TiO_2_. For 1:1 C, N–TiO_2_@ACG, Ti, O, C, and N elements were evenly distributed (Figure 3d) and specifically, Ti, O, and N were distributed in the same areas, indicating that C, N–TiO_2_ was successfully loaded on the porous skeleton of coffee grounds-derived carbon.

**Figure 4:**
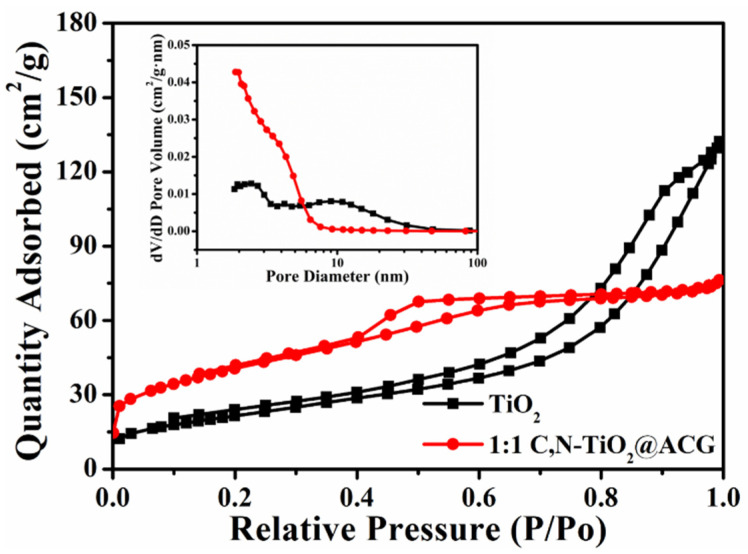
Nitrogen adsorption–desorption isotherms and pore size distribution curves (inset) of TiO_2_ and 1:1 C, N–TiO_2_@ACG.

Considering that the photocatalytic reaction occurs on the surface of the photocatalysts, the specific surface area (SSA) is a very significant parameter, which will influence the active site of the photocatalyst and its contact with pollutants, thus affecting the photocatalytic degradation efficiency. The N_2_ adsorption–desorption isotherms shown in Figure 4 present the fact that TiO_2_ and 1:1 C, N–TiO_2_@ACG exhibit type-IV isotherms, implying the existence of mesopores, which can also be directly observed from the inserted pore size distribution diagram. Compared with TiO_2_, 1:1 C, N–TiO_2_@ACG has significantly more micro- and mesopores within 10 nm, which will increase the specific surface area of the catalyst. Accordingly, the calculated SSA of the TiO_2_ and 1:1 C, N–TiO_2_@ACG is 78.7 and 145.8 m^2^/g. The loose porous structure and increased SSA of 1:1 C, N–TiO_2_@ACG will facilitate the contact and adsorption, together with the subsequent photocatalytic degradation of organic pollutants. 

**Figure 5:**
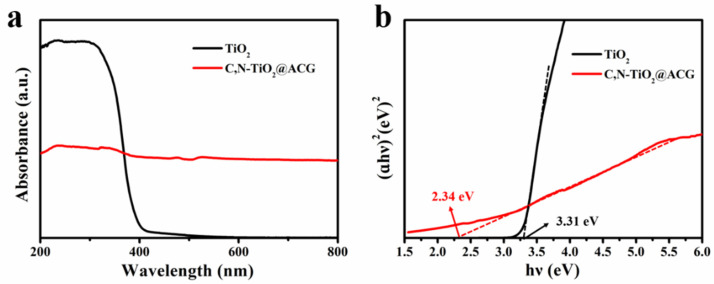
(**a**) UV–vis DRS and (**b**) band gap determined from the (ɑhυ)^2^ versus (hυ) plots for TiO_2_ and 1:1 C, N–TiO_2_@ACG.

The DRS were investigated to intuitively explore the light absorption properties and band structure of the photocatalysts and are shown in Figure 5a. TiO_2_ displays strong UV light absorption but is bare of any visible-light absorption. Notably, C, N–TiO_2_@ACG exhibits a redshift, showing a broadened spectral response range and indiscriminate absorption in both visible-light and UV-light regions, which is attributed to the C, N doping and its loading on coffee grounds-derived carbon, although the absorption intensity of UV light is inferior to that of TiO_2_. Figure 5b shows the corresponding band gap energies (Eg) calculated by the Kubelka–Munk method for TiO_2_ and 1:1 C, N–TiO_2_@ACG, which are about 3.31 and 2.34 eV, respectively. The greatly reduced Eg is because that C, N co–doping introduces impurity energy levels above the valence band of TiO_2_, narrows the band gap, and expands the response range of the catalyst to visible light [60].

**Figure 6:**
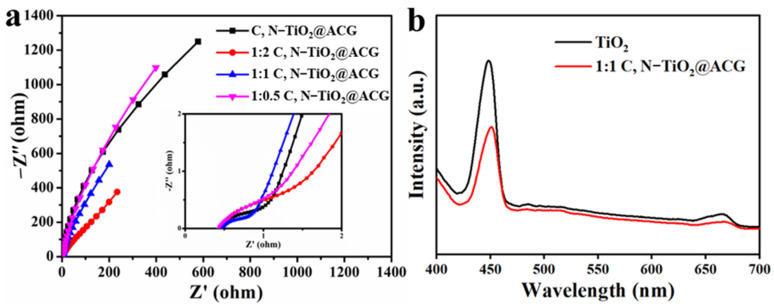
(**a**) EIS plots of photocatalysts and (**b**) PL spectra excited at a wavelength of 380 nm.

EIS was employed to identify the charge transportation dynamics of as-prepared photocatalysts and the obtained results are shown in Figure 6. Among them, 1:1 C, N–TiO_2_@ACG has the smallest Nyquist semicircle diameter, namely the lowest charge transfer resistance, indicating that the photogenerated electrons can be separated and transferred rapidly and that their recombination can be suppressed, thus increasing the effective carrier number and improving the photocatalytic activity. The PL spectrum shown in Figure 6b demonstrates an emission peak at about 450 nm for both TiO_2_ and 1:1 C, N–TiO_2_@ACG, which is consistent with previously reported TiO_2_–based photocatalysts [11], and the PL intensity of 1:1 C, N–TiO_2_@ACG was distinctly lower than that of TiO_2_, indicating the efficiently promoted separation of photo-generated electron-holes pairs, thereby also foreboding the enhanced photocatalytic activity of 1:1 C, N–TiO_2_@ACG.

**Figure 7:**
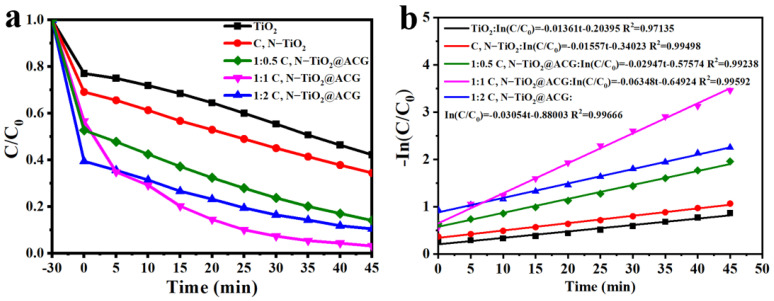
(**a**) the photocatalytic degradation rate towards MB within 45 min (**b**) the corresponding fitted photocatalytic kinetic curves (−ln (C/C_0_) = *k*t) of photocatalysts.

The photocatalytic degradation activities of as-prepared photocatalysts were investigated towards the MB under the stirring rate of 300 rpm (to simulate the actual industrial wastewater treatment), and the results are shown in Figure 7. The C/C_0_ at the time of 0 demonstrate that with the increase in ACG, the adsorption of the MB increases, directly proving the porosity and absorbability of the ACG. The characteristic absorption peak of the MB is located at the wavelength between 400 and 800 nm. After irradiation for 45 min, the photocatalytic properties follow the order of TiO_2_ (57.8%) < C, N–TiO_2_ (65.5%) < 1:0.5 C, N–TiO_2_@ACG (64.4%) < 1:2 C, N–TiO_2_@ACG (85.9%) < 1:1 C, N–TiO_2_@ACG (96.9%) (as shown in Figure 7a). To quantitatively characterize the degradation kinetics of the MB by different photocatalysts, the kinetic plots (ln (C/C_0_)) as a function of irradiation time were outlined and present a good linear fitting (Figure 7b). k of 1:1 C, N–TiO_2_@ACG is as high as 0.06348 min^−1^, which is approximately 4.66, 4.08, 2.15 and 2.08 times that of TiO_2_ (0.01361 min^−1^), C, N–TiO_2_ (0.01557 min^−1^), 1:0.5 C, N–TiO_2_@ACG (0.02947 min^−1^) and 1:2 C, N–TiO_2_@ACG (0.03054 min^−1^). These results indicate that C, N doping can partially enhance the photocatalytic performances, and for introducing the ACG substrate, too much ACG brings in more adsorption but meanwhile fewer photocatalysts, and too much TiO_2_ will cause agglomeration, thus affecting the catalytic ability. As a consequence, 1:1 C, N–TiO_2_@ACG demonstrates the optimal photocatalytic degradation capability towards the MB, which is close to or superior to the reported results, as shown in Table 1.

**Figure 8:**
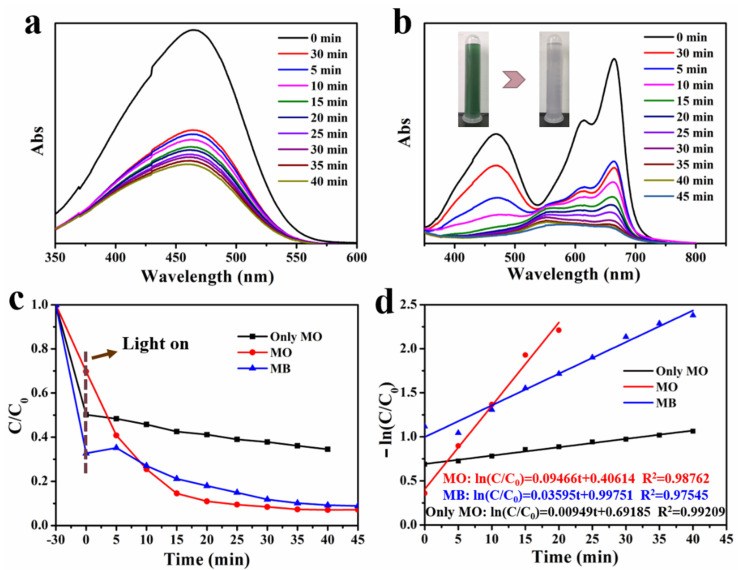
(**a**) Changes in the absorbance of degraded MO within 45 min by 1:1 C, N–TiO_2_@ACG, (**b**) Changes in absorbance of degraded MO–MB mixed solution, (**c**) the degradation rate towards MO and MB in single MO solution and MO–MB mixed solution, (**d**) the corresponding fitted photocatalytic kinetic curves.

The photocatalytic degradation of 1:1 C, N–TiO_2_@ACG towards the MO was conducted to test its catalytic ability for different dyes under simulated visible light. As shown in Figure 8a, 1:1 C, N–TiO_2_@ACG also exhibits good adsorption of the MO, and the degradation rate of the MO was 65% within 40 min. For the mixed solution of the MB and MO, the degradation effect of 1:1 C, N–TiO_2_@ACG photocatalyst was greatly enhanced, and the degradation rate of the MO already reached 90% at 20 min and more than 93% within 40 min (Figure 8b,c). The photocatalytic degradation rate k for the MO in the mixed solution was 0.09466 min^−1^, 9.97 times higher than that (0.00949 min^−1^) of the MO solution (Figure 8d). This greatly enhanced photocatalytic efficiency towards the MO in the presence of the MB is consistent with previous literature [67,68] and also indicates a possible competition between different dyes during the catalytic procedure and the mutual influence (promotion or suppression) dyes have on each other’s degradation rate depending on the specific dye. Meanwhile, 1:1 C, N–TiO_2_@ACG @ACG also had a good removal effect on the MB from mixed dye solutions, with 92% MB removal in 45 min and a photocatalytic rate of k = 0.03595 min^−1^. After 45 min of photocatalytic degradation by 1:1 C, N–TiO_2_@ACG, the mixed solution became obviously clear (as shown in the inset in Figure 8b), indicating that 1:1 C, N–TiO_2_@ACG demonstrated excellent photocatalytic capability for both single and mixed dye solutions.

**Figure 9:**
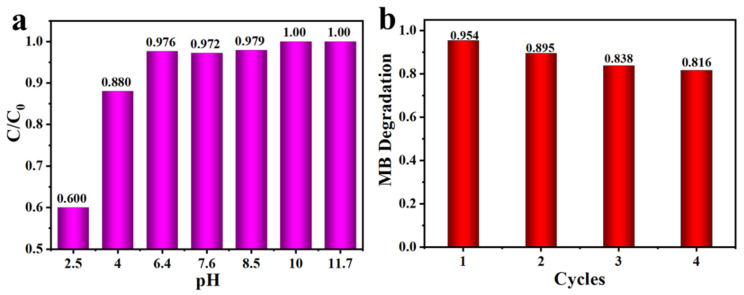
(**a**) The degradation rates of MB by 1:1 C, N–TiO_2_@ACG photocatalyst at various pH, (**b**) stability test of 1:1 C, N–TiO_2_@ACG for the degradation of MB.

The photocatalytic degradation of 1:1 C, N–TiO_2_@ACG towards the MB was operated under different pH values as shown in Figure 9a with the HCl and NaOH solution to adjust the pH value. The MB can remain stable at different pH ranges, especially in acidic conditions, possibly due to its cationic characteristics. At the pH value of less than 6.4, the degradation rate of the MB decreased significantly, being 60% at a pH of 2.5. The degradation rate became higher when the pH increased, and the MB was completely degraded within 30 min at pH = 10, and within 20 min at pH = 11.7. This trend is consistent with that of previous literature [69] and is possibly due to more hydroxyl (•OH) active radicals being generated and a strong attraction between the cationic MB and the photocatalyst under strong alkaline conditions, resulting in the enhanced degradation effect. The stability of 1:1 C, N–TiO_2_@ACG was examined through three successive cycling experiments of MB degradation. As presented in Figure 9b, 1:1 C, N–TiO_2_@ACG still maintained an over 80% removal rate for MB degradation after four recycles, demonstrating its excellent reusability and practicability.

**Figure 10:**
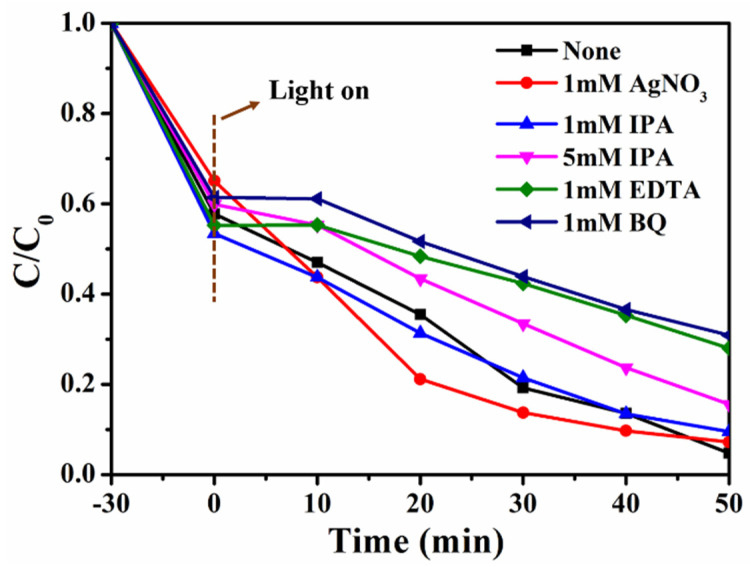
Effects of radical scavengers on the photodegradation of MB by 1:1 C, N–TiO_2_@ACG.

To better understand the possible photocatalytic degradation mechanisms, free radical trapping experiments were performed. Herein, silver nitrate (AgNO_3_), isopropanol (IPA), ethylene diamine tetraacetic acid (EDTA) and benzoquinone (BQ) were added as specific trappers of e^−^, •OH, h^+^ and •O^2−^, respectively and the corresponding results are illustrated in Figure 10. As can be seen, the presence of all trappers reduced the degradation efficiency, and the addition of BQ and EDTA greatly inhibited the photocatalytic activity, indicating that •O^2−^ and h^+^ radicals play a major role in the photocatalytic degradation process.

**Figure 11:**
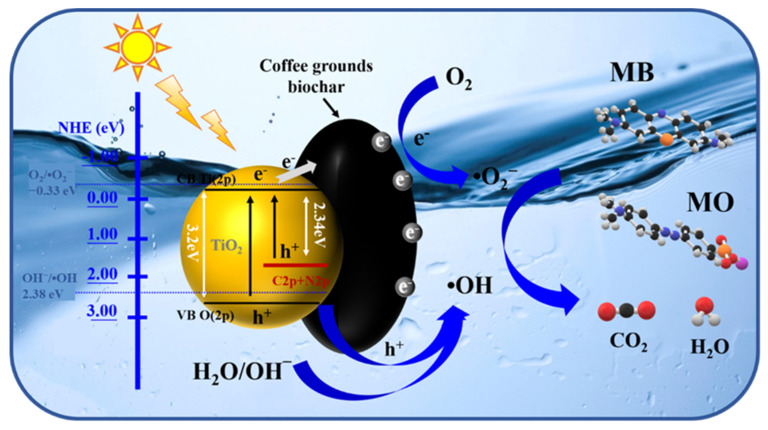
Schematic illustration of the proposed photocatalytic mechanism of 1:1 C, N-TiO_2_@ACG towards MB and MO.

Based on the aforementioned analyses, a possible degradation mechanism of 1:1 C, N–TiO_2_@ACG towards organic dyes was proposed, as shown in Figure 11. ACG possesses a porous structure and increases the specific surface area, which facilitates light absorbance and contact with pollution. Furthermore, 1:1 C, N–TiO_2_@ACG has a narrowed band gap. After irradiation, the electrons in the valence band (VB) of TiO_2_ will absorb photon energy from solar radiation and electron (e^−^) hole (h^+^) pairs in the CB and VB are excited, respectively. Normally, e^−^ and h^+^ will readily recombine, leading to low activity. Here, ACG can transfer the photogenerated electrons and suppress the recombination rate. e^−^ can easily reduce the dissolved oxygen in water to form •O_2_^−^, and accordingly, holes can also oxidize OH^−^ and H_2_O molecules adsorbed on the surface of TiO_2_ to produce •OH. Therefore, under the co-function of h^+^, •O_2_^−^ and •OH, organic dyes such as MB and MO molecules were degraded to CO_2_ and H_2_O.

## 4. Conclusions

In summary, spent coffee grounds-derived carbon that supported C, N doped TiO_2_ (C, N–TiO_2_@ACG) composite photocatalysts were prepared. Herein, spent coffee grounds-derived carbon can provide attachment sites for TiO_2_, increase contact with pollutants, and suppress the recombination of electron-hole pairs. Furthermore, C, N co–doping extends the light absorption region of TiO_2_ to the visible region, which greatly improves the photocatalytic activity. Correspondingly, the band gap energy of C, N–TiO_2_@ACG decreases from 3.31 eV of TiO_2_ to 2.34 eV. Consequently, the optimal 1:1 C, N–TiO_2_@ACG delivers remarkable photocatalytic activity with the degradation efficiency of MB reaching 96.9% within 45 min, as well as a high reaction rate of 0.06348 min^−1^, approximately 4.66 times that of TiO_2_ (0.01361 min^−1^). Furthermore, it also demonstrated greatly enhanced photocatalytic efficiency towards the MO in the presence of the MB compared with the single MO solution. In addition, it demonstrated superior degradation performance towards the MB in alkaline conditions. This work provides a reference and extension method for the further development of TiO_2_–based photocatalysts and the high value-added utilization of coffee grounds.

## Figures and Tables

**Table 1:** Degradation of MB by TiO_2_ based photocatalyst.

Photocatalysts	Irradiation	Degradation Activity	Ref
GQDs–TiO_2_	visible light	99.39%@60 min 98%@40 min	[61]
Pd–doped TiO_2_	UV	85.9%@180 min	[62]
TiO_2_–MoS_2_	UV	100%@80 min, 0.040 min^−1^	[63]
Fe–doped TiO_2_/zeolite	UV (254 nm)	98%@60 min	[64]
Ag–doped TiO_2_	visible light	96%@50 min	[65]
SiO_2_–TiO_2_	sunlight	98%@120 min	[66]
**C, N–TiO_2_@ACG**	**simulated light**	**96.9%@45 min, 0.06348 min^−1^**	**This work**

## Data Availability

The raw/processed data required to reproduce these findings cannot be shared at this time due to legal or ethical reasons.

## Supplementary Materials

The following supporting information can be downloaded at: https://www.mdpi.com/article/10.3390/ma16145137/s1, Figure S1: SEM images of (a) 1:0.5 C, N-TiO_2_@ACG and (b) 1:2 C, N-TiO_2_@ACG.

## References

[1] Xia Y., Xu L., Peng J., Han J., Guo S., Zhang L., Han Z., Komarneni S. (2019). TiO2@g-C3N4 core/shell spheres with uniform mesoporous structures for high performance visible-light photocatalytic application. Ceram. Int..

[2] Natarajan S., Bajaj H.C., Tayade R.J. (2018). Recent advances based on the synergetic effect of adsorption for removal of dyes from waste water using photocatalytic process. J. Environ. Sci..

[3] Patil S.M., Deshmukh S.P., More K.V., Shevale V.B., Mullani S.B., Dhodamani A.G., Delekar S.D. (2019). Sulfated TiO2/WO3 nanocomposite: An efficient photocatalyst for degradation of Congo red and methyl red dyes under visible light irradiation. Mater. Chem. Phys..

[4] Dai Z., Ren P.-G., Zhang H., Gao X., Jin Y.-L. (2021). Nitrogen-doped and hierarchically porous carbon derived from spent coffee ground for efficient adsorption of organic dyes. Carbon Lett..

[5] Huo M.X., Jin Y.L., Sun Z.F., Ren F., Pei L., Ren P.G. (2021). Facile synthesis of chitosan-based acid-resistant composite films for efficient selective adsorption properties towards anionic dyes. Carbohydr. Polym..

[6] Ji L., Zhang H.N., Ding W., Song R.Q., Han Y., Yu H.Y., Paneth P. (2023). Theoretical Kinetic Isotope Effects in Establishing the Precise Biodegradation Mechanisms of Organic Pollutants. Environ. Sci. Technol..

[7] Rene E.R., Kennes C., Nghiem L.D., Varjani S. (2022). New insights in biodegradation of organic pollutants Preface. Bioresour. Technol..

[8] Werber J.R., Osuji C.O., Elimelech M. (2016). Materials for next-generation desalination and water purification membranes. Nat. Rev. Mater..

[9] Wei J.-S., Song T.-B., Zhang P., Niu X.-Q., Chen X.-B., Xiong H.-M. (2020). A new generation of energy storage electrode materials constructed from carbon dots. Mater. Chem. Front..

[10] Chen X., Sun H., Zelekew O.A., Zhang J., Guo Y., Zeng A., Kuo D.-H., Lin J. (2020). Biological renewable hemicellulose-template for synthesis of visible light responsive sulfur-doped TiO_2_ for photocatalytic oxidation of toxic organic and As(III) pollutants. Appl. Surf. Sci..

[11] Jin Y., Tang W., Wang J., Ren F., Chen Z., Sun Z., Ren P.-G. (2023). Construction of biomass derived carbon quantum dots modified TiO2 photocatalysts with superior photocatalytic activity for methylene blue degradation. J. Alloys Compd..

[12] Martins A.C., Cazetta A.L., Pezoti O., Souza J.R.B., Zhang T., Pilau E.J., Asefa T., Almeida V.C. (2017). Sol-gel synthesis of new TiO2/activated carbon photocatalyst and its application for degradation of tetracycline. Ceram. Int..

[13] Mu R., Ao Y., Wu T., Wang C., Wang P. (2020). Synergistic effect of molybdenum nitride nanoparticles and nitrogen-doped carbon on enhanced photocatalytic hydrogen evolution performance of CdS nanorods. J. Alloys Compd..

[14] Ye S., Yan M., Tan X., Liang J., Zeng G., Wu H., Song B., Zhou C., Yang Y., Wang H. (2019). Facile assembled biochar-based nanocomposite with improved graphitization for efficient photocatalytic activity driven by visible light. Appl. Catal. B Environ..

[15] Shirmardi A., Teridi M.A.M., Azimi H.R., Basirun W.J., Jamali-Sheini F., Yousefi R. (2018). Enhanced photocatalytic performance of ZnSe/PANI nanocomposites for degradation of organic and inorganic pollutants. Appl. Surf. Sci..

[16] Katsumata H., Molla M.A.I., Islam J.B., Tateishi I., Furukawa M., Kaneco S. (2022). Dual Z-scheme heterojunction g-C3N4/Ag3PO4/AgBr photocatalyst with enhanced visible-light photocatalytic activity. Ceram. Int..

[17] Feng X., Wang P., Hou J., Qian J., Wang C., Ao Y. (2018). Oxygen vacancies and phosphorus codoped black titania coated carbon nanotube composite photocatalyst with efficient photocatalytic performance for the degradation of acetaminophen under visible light irradiation. Chem. Eng. J..

[18] Ouyang F., Li H., Gong Z., Pang D., Qiu L., Wang Y., Dai F., Cao G., Bharti B. (2020). Photocatalytic degradation of industrial acrylonitrile wastewater by F-S-Bi-TiO_2_ catalyst of ultrafine nanoparticles dispersed with SiO_2_ under natural sunlight. Sci. Rep..

[19] Sedaghati N., Habibi-Yangjeh A., Pirhashemi M., Vadivel S. (2019). Boosted visible-light photocatalytic performance of TiO2-x decorated by BiOI and AgBr nanoparticles. J. Photochem. Photobiol. A Chem..

[20] Jin Y., Tang W., Wang J., Chen Z., Ren F., Sun Z., Wang F., Ren P. (2022). High photocatalytic activity of spent coffee grounds derived activated carbon-supported Ag/TiO2 catalyst for degradation of organic dyes and antibiotics. Colloids Surf. A Physicochem. Eng. Asp..

[21] Schneider J., Matsuoka M., Takeuchi M., Zhang J.L., Horiuchi Y., Anpo M., Bahnemann D.W. (2014). Bahnemann, Understanding TiO2 Photocatalysis: Mechanisms and Materials. Chem. Rev..

[22] Abdel-Wahed M.S., El-Kalliny A.S., Badawy M.I., Attia M.S., Gad-Allah T.A. (2020). Core double-shell MnFe2O4@rGO@TiO2 superparamagnetic photocatalyst for wastewater treatment under solar light. Chem. Eng. J..

[23] Zhao J., Wang J., Fan L., Pakdel E., Huang S., Wang X. (2017). Immobilization of titanium dioxide on PAN fiber as a recyclable photocatalyst via co-dispersion solvent dip coating. Text. Res. J..

[24] Wen C., Zhu Y.-J., Kanbara T., Zhu H.-Z., Xiao C.-F. (2009). Effects of I and F codoped TiO2 on the photocatalytic degradation of methylene blue. Desalination.

[25] Wang J., Wang Z., Zhao D., Liang Y., Wang H., Wang N., Jiang W., Liu S., Liu C., Ding W. (2021). Preparation, structural and photocatalytic activity of Sn/Fe co-doped TiO2 nanoparticles by sol-gel method. Ceram. Int..

[26] Lei X.F., Xue X.X., Yang H., Chen C., Li X., Niu M.C., Gao X.Y., Yang Y.T. (2015). Effect of calcination temperature on the structure and visible-light photocatalytic activities of (N, S and C) co-doped TiO_2_ nano-materials. Appl. Surf. Sci..

[27] Fahim S.A., Zahan N., Shathy R.A., Quddus M.S., Moniruzzaman M., Masum S.M., Molla M.A.I. (2023). B–Sn/TiO_2_ nanoparticles for photodegradation of metronidazole antibiotics under different lights. Mater. Chem. Phys..

[28] Qu G., Wang H., Li X., Wang T., Zhang Z., Liang D., Qiang H. (2021). Enhanced removal of acid orange II from aqueous solution by V and N co-doping TiO_2_-MWCNTs/gamma-Al_2_O_3_ composite photocatalyst induced by pulsed discharge plasma. Water Sci. Technol..

[29] Gong S., Fan J., Cecen V., Huang C., Min Y., Xu Q., Li H. (2021). Noble-metal and cocatalyst free W_2_N/C/TiO photocatalysts for efficient photocatalytic overall water splitting in visible and near-infrared light regions. Chem. Eng. J..

[30] Tu B., Chen H., Deng J., Xue S., Ma X., Xu Y., Xie Z., Tao H. (2021). Preparation of N-I co-doped TiO2 supported on activated carbon photocatalyst for efficient photocatalytic reduction of Cr(Ⅵ) ions. Colloids Surf. A Physicochem. Eng. Asp..

[31] Delgado-Díaz D., Hernández-Ramírez A., Guzmán-Mar J.L., Villanueva-Rodríguez M., Maya-Treviño L., Hinojosa-Reyes L. (2021). N-S co-doped TiO2 synthesized by microwave precipitation method: Effective photocatalytic performance for the removal of organoarsenic compounds. J. Environ. Chem. Eng..

[32] Asahi R., Morikawa T., Ohwaki T., Aoki K., Taga Y. (2001). Visible-Light Photocatalysis in Nitrogen-Doped Titanium Oxides. Science.

[33] Zhang Y., Han C., Nadagouda M.N., Dionysiou D.D. (2015). The fabrication of innovative single crystal N,F-codoped titanium dioxide nanowires with enhanced photocatalytic activity for degradation of atrazine. Appl. Catal. B Environ..

[34] Komatsuda S., Asakura Y., Vequizo J.J.M., Yamakata A., Yin S. (2018). Enhanced photocatalytic NO decomposition of visible-light responsive F-TiO_2_/(N,C)-TiO_2_ by charge transfer between F-TiO_2_ and (N,C)-TiO_2_ through their doping levels. Appl. Catal. B Environ..

[35] Asahi R., Morikawa T., Irie H., Ohwaki T. (2014). Nitrogen-Doped Titanium Dioxide as Visible-Light-Sensitive Photocatalyst: Designs, Developments, and Prospects. Chem. Rev..

[36] Di Valentin C., Pacchioni G. (2013). Trends in non-metal doping of anatase TiO_2_: B, C, N and F. Catal. Today.

[37] Livraghi S., Paganini M.C., Giamello E., Selloni A., Di Valentin C., Pacchioni G. (2006). Origin of photoactivity of nitrogen-doped titanium dioxide under visible light. J. Am. Chem. Soc..

[38] Mollavali M., Falamaki C., Rohani S. (2018). Efficient light harvesting by NiS/CdS/ZnS NPs incorporated in C, N-co-doped-TiO2 nanotube arrays as visible-light sensitive multilayer photoanode for solar applications. Int. J. Hydrogen Energy.

[39] Lin Y.-T., Weng C.-H., Lin Y.-H., Shiesh C.-C., Chen F.-Y. (2013). Effect of C content and calcination temperature on the photocatalytic activity of C-doped TiO2 catalyst. Sep. Purif. Technol..

[40] Han X., An L., Hu Y., Li Y., Hou C., Wang H., Zhang Q. (2020). Ti_3_C_2_ MXene-derived carbon-doped TiO_2_ coupled with g-C_3_N_4_ as the visible-light photocatalysts for photocatalytic H_2_ generation. Appl. Catal. B Environ..

[41] Farhadian N., Akbarzadeh R., Pirsaheb M., Jen T.C., Fakhri Y., Asadi A. (2019). Chitosan modified N, S-doped TiO(2) and N, S-doped ZnO for visible light photocatalytic degradation of tetracycline. Int. J. Biol. Macromol..

[42] Kholodnaya G., Sazonov R., Ponomarev D. (2021). TiO_2_@C nanocomposites—From synthesis to application: A review. Fuller. Nanotub. Carbon Nanostruct..

[43] Karthikeyan K.T., Nithya A., Jothivenkatachalam K. (2017). Photocatalytic and antimicrobial activities of chitosan-TiO_2_ nanocomposite. Int. J. Biol. Macromol..

[44] Shao Y., Cao C., Chen S., He M., Fang J., Chen J., Li X., Li D. (2015). Investigation of nitrogen doped and carbon species decorated TiO_2_ with enhanced visible light photocatalytic activity by using chitosan. Appl. Catal. B Environ..

[45] Habibi-Yangjeh A., Feizpoor S., Seifzadeh D., Ghosh S. (2020). Improving visible-light-induced photocatalytic ability of TiO_2_ through coupling with Bi_3_O_4_Cl and carbon dot nanoparticles. Sep. Purif. Technol..

[46] Feizpoor S., Habibi-Yangjeh A., Ahadzadeh I., Yubuta K. (2019). Oxygen-rich TiO_2_ decorated with C-Dots: Highly efficient visible-light-responsive photocatalysts in degradations of different contaminants. Adv. Powder Technol..

[47] Zhang H., Lv X., Li Y., Wang Y., Li J. (2010). P25-Graphene Composite as a High Performance Photocatalyst. Acs Nano.

[48] Tokimoto T., Kawasaki N., Nakamura T., Akutagawa J., Tanada S. (2005). Removal of lead ions in drinking water by coffee grounds as vegetable biomass. J. Colloid. Interface Sci..

[49] Xiao F., Guo X., Li J., Sun H., Zhang H., Wang W. (2019). Electrospinning preparation and dye adsorption capacity of TiO_2_@Carbon flexible fiber. Ceram. Int..

[50] Qin J., Chen Q., Sun M., Sun P., Shen G. (2017). Pyrolysis temperature-induced changes in the catalytic characteristics of rice husk-derived biochar during 1,3-dichloropropene degradation. Chem. Eng. J..

[51] Chatterjee S., Saito T. (2015). Lignin-Derived Advanced Carbon Materials. ChemSusChem.

[52] Wang Z., Smith A.T., Wang W., Sun L. (2018). Versatile Nanostructures from Rice Husk Biomass for Energy Applications. Angew. Chem. Int. Ed. Engl..

[53] Shi M., Wei W., Jiang Z., Han H., Gao J., Xie J. (2016). Biomass-derived multifunctional TiO_2_/carbonaceous aerogel composite as a highly efficient photocatalyst. RSC Adv..

[54] Saratale G.D., Bhosale R., Shobana S., Banu J.R., Pugazhendhi A., Mahmoud E., Sirohi R., Bhatia S.K., Atabani A.E., Mulone V. (2020). A review on valorization of spent coffee grounds (SCG) towards biopolymers and biocatalysts production. Bioresour. Technol..

[55] Ferraz F.M., Yuan Q. (2020). Organic matter removal from landfill leachate by adsorption using spent coffee grounds activated carbon. Sustain. Mater. Technol..

[56] Pagalan E., Sebron M., Gomez S., Salva S.J., Ampusta R., Macarayo A.J., Joyno C., Ido A., Arazo R. (2020). Activated carbon from spent coffee grounds as an adsorbent for treatment of water contaminated by aniline yellow dye. Ind. Crops Prod..

[57] Guo Z., Ren P., Zhang Z., Dai Z., Lu Z., Jin Y., Ren F. (2022). Fabrication of carbonized spent coffee grounds/graphene nanoplates/cyanate ester composites for superior and highly absorbed electromagnetic interference shielding performance. J. Mater. Sci. Technol..

[58] He W., Ren P.-G., Dai Z., Hou X., Ren F., Jin Y.-L. (2021). Hierarchical porous carbon composite constructed with 1-D CNT and 2-D GNS anchored on 3-D carbon skeleton from spent coffee grounds for supercapacitor. Appl. Surf. Sci..

[59] Chandrabose G., Dey A., Gaur S.S., Pitchaimuthu S., Jagadeesan H., Braithwaite N.S.J., Selvaraj V., Kumar V., Krishnamurthy S. (2021). Removal and degradation of mixed dye pollutants by integrated adsorption-photocatalysis technique using _2_-D MoS_2_/TiO_2_ nanocomposite. Chemosphere.

[60] Papailias I., Todorova N., Giannakopoulou T., Dvoranová D., Brezová V., Dimotikali D., Trapalis C. (2021). Selective removal of organic and inorganic air pollutants by adjusting the g-C3N4/TiO2 ratio. Catal. Today.

[61] Rawal J., Kamran U., Park M., Pant B., Park S.-J. (2022). Nitrogen and Sulfur Co-Doped Graphene Quantum Dots Anchored TiO2 Nanocomposites for Enhanced Photocatalytic Activity. Catalysts.

[62] Nguyen C.H., Fu C.-C., Juang R.-S. (2018). Degradation of methylene blue and methyl orange by palladium-doped TiO2 photocatalysis for water reuse: Efficiency and degradation pathways. J. Clean. Prod..

[63] Ibukun O., Evans P.E., Dowben P.A., Jeong H.K. (2019). Titanium dioxide-molybdenum disulfide for photocatalytic degradation of methylene blue. Chem. Phys..

[64] Foura G., Chouchou N., Soualah A., Kouachi K., Guidotti M., Robert D. (2017). Fe-Doped TiO_2_ Supported on HY Zeolite for Solar Photocatalytic Treatment of Dye Pollutants. Catalysts.

[65] Skiba M., Vorobyova V. (2020). Synthesis OF AG/TIO2 nanocomposite via plasma liquid interactions and degradation methylene blue. Appl. Nanosci..

[66] Mahanta U., Khandelwal M., Deshpande A.S. (2022). TiO_2_@SiO_2_ nanoparticles for methylene blue removal and photocatalytic degradation under natural sunlight and low-power UV light. Appl. Surf. Sci..

[67] Motelica L., Vasile B.S., Ficai A., Surdu A.V., Ficai D., Oprea O.C., Andronescu E., Jinga D.C., Holban A.M. (2022). Influence of the Alcohols on the ZnO Synthesis and Its Properties: The Photocatalytic and Antimicrobial Activities. Pharmaceutics.

[68] Motelica L., Oprea O.C., Vasile B.S., Ficai A., Ficai D., Andronescu E., Holban A.M. (2023). Antibacterial Activity of Solvothermal Obtained ZnO Nanoparticles with Different Morphology and Photocatalytic Activity against a Dye Mixture: Methylene Blue, Rhodamine B and Methyl Orange. Int. J. Mol. Sci..

[69] Sathiyan K., Bar-Ziv R., Mendelson O., Zidki T. (2020). Controllable synthesis of TiO_2_ nanoparticles and their photocatalytic activity in dye degradation. Mater. Res. Bull..

