# Supplementary figures and images for: Spent Coffee Grounds Derived Carbon Loading C, N Doped TiO2 for Photocatalytic Degradation of Organic Dyes
Source: Materials (Basel). 2023 Jul 21;16(14):5137. doi: 10.3390/ma16145137 (PMC10385829; doi:10.3390/ma16145137)

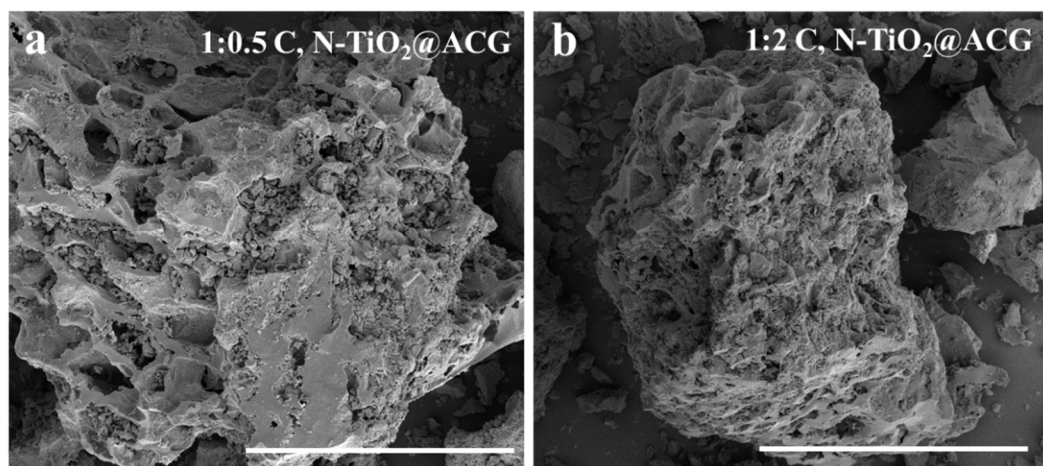

**Figure S1.** SEM images of (a) 1:0.5 C, N-TiO<sub>2</sub>@ACG and (b) 1:2 C, N-TiO<sub>2</sub>@ACG.

Supplement: Supplementary file 1 [file materials-16-05137-s001.zip › materials-2486762-supplementary.pdf]
